# Effectiveness of Sequential Viscosupplementation in Temporomandibular Joint Internal Derangements and Symptomatology: A Case Series

**DOI:** 10.1155/2018/5392538

**Published:** 2018-07-31

**Authors:** Roberta Maria Drumond Furtado Bossi Fonseca, Eduardo Januzzi, Luciano Ambrosio Ferreira, Eduardo Grossmann, Antonio Carlos Pires Carvalho, Pedro Gonçalves de Oliveira, Érica Leandro Marciano Vieira, Antônio Lúcio Teixeira, Camila Megale Almeida-Leite

**Affiliations:** ^1^Programa de Pós-Graduação em Patologia, Faculdade de Medicina, Universidade Federal de Minas Gerais (UFMG), Belo Horizonte, MG, Brazil; ^2^Faculdade Ciodonto, Sete Lagoas, MG, Brazil; ^3^Departamento de Radiologia, Faculdade de Medicina, Universidade Federal do Rio de Janeiro, Rio de Janeiro, RJ, Brazil; ^4^9 Hospital Maternidade Therezinha de Jesus-HMTJ/JF, Suprema-Faculdade de Ciências Médicas e da Saúde, Juiz de Fora, Brazil; ^5^Departamento de Ciências Morfológicas, Instituto de Ciências Básicas da Saúde, Universidade Federal do Rio Grande do Sul, Porto Alegre, RS, Brazil; ^6^Faculdade de Farmácia, Universidade Anhembi Morumbi, São Paulo, SP, Brazil; ^7^Departamento de Clínica Médica, Faculdade de Medicina, UFMG, Belo Horizonte, MG, Brazil; ^8^Department of Psychiatry and Behavioral Sciences, The University of Texas, Houston, TX, USA; ^9^Departamento de Morfologia, Instituto de Ciências Biológicas, UFMG, Belo Horizonte, MG, Brazil

## Abstract

Viscosupplementation is a minimally invasive technique that replaces synovial fluid by intra-articular injection of hyaluronic acid (HA). Although effective in some joints, there is not conclusive evidence regarding temporomandibular disorders. This case series described the efficacy of a viscosupplementation protocol in intra-articular temporomandibular disorders. Ten patients with a diagnosis of disc displacement and/or osteoarthritis by Research Diagnostic Criteria for Temporomandibular Disorders (RDC/TMD) were submitted to four monthly injections of low or medium molecular weight HA. Pain, mandibular function, image analysis by tomography and magnetic resonance, and quality of life were assessed at baseline and follow-ups (1 and 6 months). Pain, jaw range-of-motion, mandibular function, and quality of life improved at follow-up evaluations. Osteoarthritis changes decreased, and 20% of patients improved mandibular head excursion after treatment. Resolution of effusion and improvement in disc morphology were observed for most patients. This viscosupplementation protocol reduced pain and symptoms associated with internal derangement of temporomandibular joint, improved quality of life, and showed benefits from both low and medium molecular weight HA in alternate cycles.

## 1. Introduction

Temporomandibular disorders (TMDs) are a heterogeneous group of disorders involving the temporomandibular joint (TMJ), the masticatory muscles, and associated structures [1–3]. TMD affect 5 to 12% of population [4], and their management causes high costs to public health [5]. The most common signs and symptoms include pain, TMJ sounds, and limitation of mandibular movement, which can compromise daily activities and quality of life [2, 6].

According to American Academy of Orofacial Pain (AAOP), diagnosis and classification of TMD are divided into two major groups: muscle and joint disorders, with their respective subdivisions [3]. Among intra-articular TMD, disc displacement with or without reduction and degenerative joint disorders (osteoarthrosis and osteoarthritis) are the most frequent alterations. They are associated to changes in quantity and quality of synovial fluid (SF) [3, 7].

Viscosupplementation (VS) is a minimally invasive technique that involves replacement of synovial fluid by intra-articular injection of hyaluronic acid (HA) which restores its concentration and molecular weight in joint cavity [8]. HA is an important component of synovial fluid and is produced by type B synoviocytes. These molecules are involved by a large amount of water and provide suitable viscosity and elasticity for synovial fluid [9]. Studies about the effects of exogenous HA with different molecular weights have been performed. It has been suggested that high molecular weight HA is important in lubrication and protection of joint structures due to its improvement of highly hydrated and rheological environment [10, 11]. In contrast, low molecular weight HA induces its endogen production by type B synoviocytes restoring natural properties of synovial fluid [12, 13].

VS has been proven to be effective for knee and other large joints [14], and it can stimulate de novo synthesis of HA and inhibits release of inflammatory mediators by synoviocytes [8], such as cytokines and metalloproteinases that have been associated with osteoarthritis, mediating pain, and tissue damage [15–18].

Regarding TMD, there is not conclusive evidence [19–21]. Several studies have shown that VS can improve lubrication and biomechanical properties of TMJ and eliminate or reduce joint-related pain [22–26], but different concentrations and molecular weights of HA, varied number of intra-articular injections, and treatment cycles made it difficult to establish an effective approach [19–21]. Recent systematic reviews have shown that HA intra-articular injections in TMJ can be beneficial in improving pain and symptoms of TMDs and in regulating inflammatory mediators better than placebo, but they highlight that further clinical research is necessary to establish its effectiveness, mainly in comparison to corticosteroid [19–21]. Moreover, these works emphasize that an adequate protocol with number of injections, appropriate molecular weight of HA, minimum effective dose, and long-term side effects should be addressed [19–21].

Based upon clinical use of VS in joint disorders, including TMD, and the need of an efficient protocol for treatment, we describe a case series of four monthly injections of low and medium molecular weight HA in superior TMJ compartment and analyze TMJ dysfunction and quality of life through validated instruments and TMJ image analysis.

## 2. Materials and Methods

This study was approved by the Ethics Committee of Universidade Federal de Minas Gerais, Belo Horizonte, Brazil (CAAE-24911314.3.0000.5149) and registered in Brazilian Registry of Clinical Trials (RBR-6759yz). All procedures were performed in accordance with the ethical standards of institutional and/or national research committee and with the principles stated in the 1964 Helsinki Declaration and its later amendments. All patients provided written informed consent before inclusion in the study and received free and unconditional treatment.

Ten consecutive patients fulfilling the following inclusion criteria were selected from university orofacial pain division or from a private orofacial pain clinic: age between 18 and 70, diagnosis of disc displacement with or without reduction, and/or osteoarthritis according to the Research Diagnostic Criteria for Temporomandibular Disorders (RDC/TMD Axis I). Patients with rheumatologic diseases, neuropathic pain, or history of previous TMJ surgery, trauma, or fractures were excluded. No other treatment for TMD (physical therapy, jaw exercises, heat pack to the jaw, and muscle relaxants) was allowed during the study period, and anamnesis before each session was performed to control it.

### 2.1. Viscosupplementation

All ten selected patients underwent a cycle of four injections (1 per month) of 1 mL of HA in upper joint compartment of both joints as previously described [7]. Low MW HA (500–730 kDa, Polireumin®) was used in months 1 and 3 and medium MW HA (1,000–2,000 kDa, Osteonil Mini®) was injected in months 2 and 4. All injection procedures were conducted by the same physician. Baseline evaluation and two follow-up assessments (1 month and 6 months) were performed after the end of the treatment.

### 2.2. Clinical Evaluation

Clinical evaluations were performed by the same experienced operator after training and calibration by RDC/TMD examination protocol [27]. The following parameters were assessed at the time of diagnosis (baseline) and at each appointment during treatment (data not shown) and follow-ups (1 and 6 months after treatment): (1) pain intensity by 0–10 numeric rating scale (NRS) (0 = no pain and 10 = worst possible pain) [28]; (2) pain quality by multidimensional McGill Pain Questionnaire (MPQ), which characterizes emotional and sensory aspects of pain with scores ranging from 1 to 78 [29]; (3) pain-related impact of life by Manchester Orofacial Pain Disability Scale (MOPDS-Brazil), a 26-item Likert scale questionnaire with scores ranging from 0 to 52 [30]; (4) jaw range-of-motion by interincisal distance; (5) severity of craniomandibular dysfunction by Clinical Dysfunction Index Craniomandibular (IDCCM), ranging from 0 to 5 [31]; (6) functional limitation by Mandibular Function Impairment Questionnaire (MFIQ), a 17-item Likert scale questionnaire with final score ranging from 0 to 5 [32]; (7) quality of life by Oral Health Impact Profile (OHIP- 49) with values from 0 to 280 [33].

### 2.3. Image Analysis

The patients' left and right joints were examined by cone-beam computerized tomography (CBCT) and by magnetic resonance imaging (MRI) at baseline and at final follow-up (6 months after treatment). Images were interpreted by a blind experienced radiologist and all available slices were evaluated. In CBCT, osteoarthritic (OA) changes were defined according to Ahmad et al. [34] by the presence of sclerosis (loss of convex aspect in the articular surface), osteophyte (reactive bone spirits), erosion (cortical rupture), and subchondral cyst (pseudocyst infiltrated in the subcortical region). All parameters were analyzed in sagittal and coronal views of 1 mm interval through Radiocef Studio 2 software as previously described [34]. The distance of the outmost points of detected alterations were compared between baseline and final follow-up images in the same tomographic slice. Position of mandibular head in relation to temporal bone was assessed by visual inspection of the CBCT scan slides and categorized as normal mobility, hypomobility or hypermobility. In MRI, posterior band disc joint position in sagittal and coronal views was evaluated as previously described [18], and methods of image analysis for MRI was similar of CBCT. Presence of reduction, adhesion, and effusion (inflammatory signals) was also analyzed. In addition, morphology of disc was classified as previously described [35].

### 2.4. Statistical Analysis

Statistical analysis was performed using MINITAB® software version 17. For clinical data, within-patient differences among baseline and follow-up values were assessed by paired *t*-tests for comparing mean change or Wilcoxon signed rank test for comparing median change. Osteoarthritic changes between baseline and final follow-up (6 months) were evaluated by paired *t*-test for comparing mean change (erosion) or by Wilcoxon signed rank test for comparing median change (sclerosis, osteophyte, and flattening). *P* values of 0.05 or less were considered significant. All graphs were created by GraphPad Prism 5.0 software.

## 3. Results

Demographic characteristics (age, gender, race/ethnicity, marital status, and scholarity) of sample are shown in Table 1.

### 3.1. Clinical Evaluation

At baseline, 50% of patients (*n*=5 patients) had myofascial pain according to RDC/TMD Axis I Group I (muscle disorders) (Table 2). In RDC/TMD Axis I Group II disorders (disc displacement), 90% (*n*=9 patients) were diagnosed with disc displacement with reduction (ADDR). Whereas, in RDC/TMD Axis I Group III (other joint conditions), 10% (*n*=1 patient) had arthralgia at rest and mandibular function and 20% (*n*=2 patients) had osteoarthosis/osteoarthritis diagnosis.

One and 6 months after treatment, there was a significant change in patient diagnosis according to the RDC/TMD Axis I Group I, that is, no patient was diagnosed with myofascial pain. No changes were observed in RDC/TMD Axis I Group II, except for one patient. In RDC/TMD Axis I Group III, the patient with arthralgia became symptomless and one of the patients formerly diagnosed with osteoarthritis was diagnosed with osteoarthrosis.

**Figure 1:**
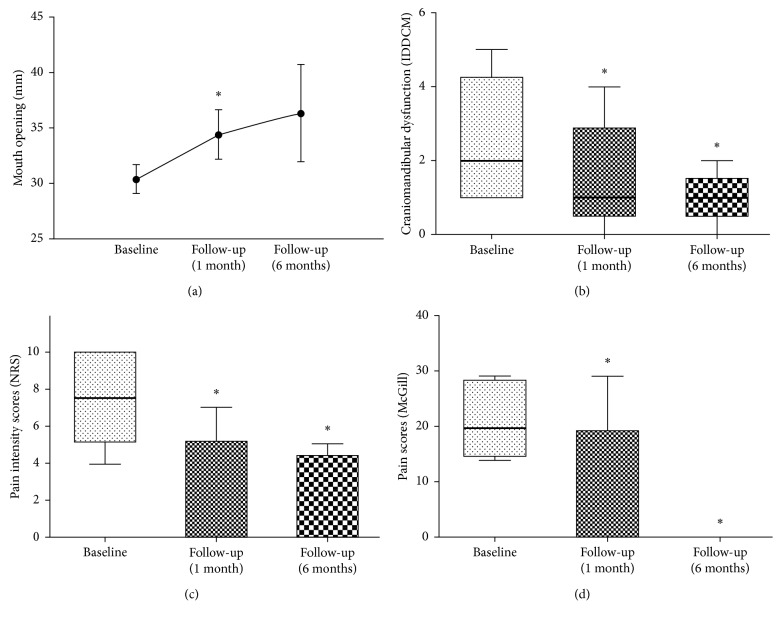
(a) Improvement on mouth opening without pain (measured in mm) at 1 and 6 months after treatment. This parameter was analyzed only on patients who showed limited mouth opening at baseline. Bars represent standard deviation (SD). Student's *t*-test; ^*∗*^*p*=0.039; *n*=5 patients (1-month follow-up) and 3 patients (6-month follow-up). (b) Decrease in scores of craniomandibular dysfunction (IDDCM-Helkimo Index) at 1 and 6 months after treatment. Box and whisker show quartiles, the band inside the box is the median, and the ends of the whiskers represent minimum and maximum values. Wilcoxon signed rank test; ^*∗*^*p*=0.034 (1-month follow-up) and ^*∗*^*p*=0.038 (6-month follow-up); *n*=10 patients (1-month follow-up) and 8 patients (6-month follow-up). (c) Decrease in NRS pain intensity at 1 and 6 months after treatment. This parameter was analyzed only on patients who showed pain at baseline. Box and whisker show quartiles, the band inside the box is the median, and the ends of the whiskers represent minimum and maximum values. Wilcoxon signed rank test; ^*∗*^*p*=0.018 (1-month follow-up) and ^*∗*^*p*=0.05 (6-month follow-up); *n*=6 patients (1-month follow-up) and 4 patients (6-month follow-up). (d) Decrease in McGill pain scores at 1 and 6 months after treatment. This parameter was analyzed only on patients who showed pain at baseline. Box and whisker show quartiles, the band inside the box is the median, and the ends of the whiskers represent minimum and maximum values. Wilcoxon signed rank test; ^*∗*^*p*=0.042 (1-month follow-up) and ^*∗*^*p*=0.05 (6-month follow-up); *n*=6 patients (1-month follow-up) and 4 patients (6-month follow-up)

Mouth opening without pain improved at 1 month after treatment in comparison to baseline (Figure 1(a)). Craniomandibular dysfunction showed significant improvement 1 and 6 months after treatment (Figure (1(b))). Pain intensity was significantly decreased at 1 and 6 months (Figure 1(c)), as well as McGill pain scores (Figure 1(d)).

**Figure 2:**
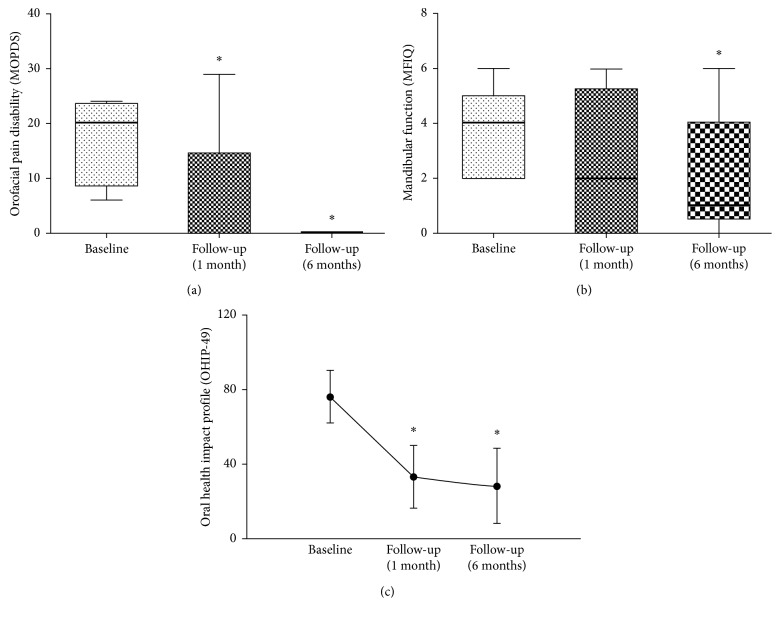
(a) Improvement on orofacial pain disability at 1 and 6 months after treatment. This parameter was analyzed only on patients who showed pain at baseline. Box and whisker show quartiles, the band inside the box is the median, and the ends of the whiskers represent minimum and maximum values. Wilcoxon signed rank test; ^*∗*^*p*=0.042 (1-month follow-up) and ^*∗*^*p*=0.05 (6-month follow-up); *n*=6 patients (1-month follow-up) and 4 patients (6-month follow-up). (b) Improvement on mandibular function MFIQ at 6 months after treatment. Box and whisker show quartiles, the band inside the box is the median, and the ends of the whiskers represent minimum and maximum values. Wilcoxon signed rank test; *p* < 0.05 (1-month follow-up) and, ^*∗*^*p*=0.038 (6-month follow-up); *n*=10 patients (1-month follow-up) and 8 patients (6-month follow-up). (c) Decrease of impact on quality of life (OHIP-49) at 1 and 6 months. Bars represent standard deviation (SD). Student's *t*-test; ^*∗*^*p*=0.029 (1-month follow-up) and ^*∗*^*p*=0.035 (6-month follow-up); *n*=10 patients (1-month follow-up) and 8 patients (6-month follow-up).

Moreover, orofacial pain disability was improved at 1 and 6 months follow-up evaluations (Figure 2(a)) and better mandibular function was detected 6 months after treatment (Figure 2(b)). Quality of life reported by patients showed improvement at both follow-up evaluations in comparison to baseline (Figure 2(c)).

### 3.2. Image Analysis

At baseline, both TMJs of all patients were examined by CBCT and MRI. Osteoarthritic changes evaluation by CBCT showed significant decrease in presence of osteophyte, flattening, sclerosis, and erosion of mandibular head at 6 months after treatment (Table 3). Hypoplasia and hyperplasia of mandibular head, deviation in form, subcortical cysts, generalized sclerosis, loose joint body or bone ankylosis were not found at baseline or 6 months follow-up. In addition, CBCT has shown that 20% of patients (2 patients) have improved standard excursion of mandibular head in both joints after treatment.

Soft tissue evaluation by MRI before and 6 months after treatment showed the following: (1) all patients had disc displacement with reduction before and after treatment; (2) all patients had alterations in disc position in at least one of the views (sagittal and/or coronal) after treatment; (3) one patient showed remission of right disc adhesion after treatment; (4) all patients (4 joints) who had effusion signal before treatment evolved to resolution of effusion 6 months after treatment (Table 4). Regarding disc shape, all patients showed stabilization or improvement in disc morphology of both joints, except for one patient.

## 4. Discussion

In this case series, we evaluated the effectiveness of a protocol of four injections of low and medium MW HA on pain, mandibular function, signs of intra-articular disease by image analysis, and quality of life in ten patients with TMD.

After treatment, disc displacement diagnosis by MRI or RDC/TMD was not changed, except for one patient, which was expected since TMJ discs cannot be replaced by minimal invasive technique [36]. Disc position in coronal view was altered in 5 joints after treatment, and this may be due to better lubrication and recovery of mandibular dynamics obtained by VS. Joint sound is the clinical sign that RDC/TMD utilizes for disc displacement diagnosis, but disc position can only be determined by MRI analysis [27]. Since VS improves joint lubrication and biomechanics, joint sound may not be present even when disc is displaced. This might be the case for the patient that had a change in clinical diagnosis by RDC/TMD, although image analysis did not change.

All patients initially diagnosed with muscle pain (myofascial), joint pain (arthralgia), or limited mouth opening have improved pain and function and those diagnoses were not observed at follow-ups. Pain relief was observed by a significant reduction of pain intensity and scores measured by NRS, McGill, and MOPDS. This may be attributed to different mechanisms regarding TMJ, such as anti-inflammatory effects of HA injection with consequent decrease of metalloproteinases and proinflammatory mediators in synovial fluid, as well as improvement of joint biomechanics [10–13]. In this work, measurement of synovial fluid inflammatory mediators was not performed to avoid invasive technique of TMJ, which could create bias in treatment outcome. Moreover, masticatory muscles promote jaw movements and their functionality is related to structural and functional integrity of TMJ [2]. Hence, relief or improvement of joint symptoms, as well as restoration of biomechanics by VS protocol, may be associated with better function of adjacent muscles and pain relief. Moreover, diminished peripheral inputs by restored TMJ may lead to improvement of central sensitization and muscle pain [37].

VS protocol tested here showed significant improvement in mouth opening amplitude both in clinical and radiologic evaluations. This outcome in clinical examination has also been shown in other studies of VS but with different protocols [7, 8, 24, 25] and may be due to restoration of joint lubrication. Moreover, VS was able to improve medial disc position, shown by MRI, which may have contributed to better mandible movements, TMJ biomechanics, and quality of life.

Less severe dysfunction was observed after treatment. Evaluation of mandible function by MFIQ has also shown improvement. More importantly, patients' evaluation of quality of life has improved. Other studies have also shown beneficial outcomes of VS by mouth opening, pain intensity, and subjective parameters such as satisfaction with treatment [7, 24, 25]. However, to our knowledge, objective evaluation of TMJ dysfunction, mandible function, and quality of life through validated instruments is first described here.

It is important to highlight that pain relief as well as improvement in mouth opening, mandibular function, and quality of life may also be a result of observed remission of myofascial pain itself. As mentioned, masticatory muscles and TMJ are structurally functionally related [2]. Moreover, reduction on pain could be also attributed to a better consciousness of mandibular function or to a placebo effect as a consequence of being under of examination and treatment for TMD. However, this hypothesis cannot be tested or excluded at this time.

Only a few studies have used image analysis to evaluate TMD treatment efficacy [18, 26]. In this work, image analysis revealed positive effects of established therapeutics in shape and function of hard and soft tissues of TMJ. VS improvement of biomechanics and lubrication seems to stabilize disc shape and avoid greater deformities, which is relevant for the course of the disease [34]. Moreover, effusion signals were not observed after treatment and our VS protocol showed effectiveness in recovery of joint inflammation and OA degenerative changes. VS beneficial effects such as reduction of joint friction, improvement of rheological environment [10, 11], and induction of endogen production of HA [12, 13] may lead to anatomical rearrangement and can justify CBCT and MRI tissue remodeling observed here.

Among studies that have shown efficacy of VS in TMD, different methods have been described and, as a result, there is an effort of researchers and clinicians to establish an effective protocol for treatment of TMD, as already established for other joints [7, 12, 24, 38]. The present study shows a new protocol of four injections of low and medium MW HA in TMJ with relevant clinical effectiveness on pain, jaw range of motion, dysfunction degree, and quality of life. Furthermore, it is important to emphasize that VS as a single intra-articular treatment is less aggressive than other techniques such as arthrocentesis [7, 24], associated or not with VS, with safety and economic advantages.

The use of HA of different MW in alternated monthly injections is a new perspective of VS in TMD and allows association of biomechanical properties of high MW AH and biological effects of lower MW AH. Hence, this protocol of treatment is able to promote fast and sustained effects, as suggested by results.

The literature describes different time intervals between applications [24, 38]. We believe that 1-month interval may allow HA acting inside joint for longer periods, which favor the effects of the next injection and the treatment itself. In addition, treatment cycle with monthly injections may be more tolerated by patients and offer some economic benefits, as it postpones a new cycle. Improvement of pain, mandibular function, and quality of life are in accordance to this finding, and relief of TMD signs and symptoms offered by VS may have restored local and systemic functions.

Although we show promising results regarding the described protocol for TMJ VS, we are aware of the limitations of this work. We believe its greater contribution may be the description of a new perspective to be tested in a well-controlled clinical trial in future research studies. Our small number of patients and the study design as an open label noncontrolled trial does not allow inference of VS positive effects to all TMD patients. However, case series is a descriptive work that illustrates novel features in clinical practice, its sample represents common clinical population, and generates new research questions [39]. Hence, this study aimed at sharing a description of some well succeeded cases of sequential VS in TMJ internal derangements. Moreover, case series usually describes 5 to 7 cases [40], and our sample is in accordance to this type of work, even with loss of 2 patients at final follow-up.

VS protocol shown here reduced pain and symptoms associated with internal derangement of TMJ and improved quality of life of TMD patients. Randomized clinical trials of this treatment protocol should deserve attention in future researches.

## Figures and Tables

**Table 1:** Demographic characteristics of patients.

Patient	Age	Gender	Race/ethnicity	Marital status	Education
1	35	F	Other or unstated	Never married	High school or less
2	47	F	Other or unstated	Married	High school or less
3	34	M	Other or unstated	Married	High school or less
4	66	F	White	Married	High school or less
5	20	F	White	Never married	Undergraduate degree
6	30	F	Other or unstated	Married	High school or less
7	19	F	White	Never married	Undergraduate degree
8	27	F	Other or unstated	Never married	Postgraduate degree
9	43	F	Other or unstated	Divorced	High school or less
10	37	F	White	Never married	Postgraduate degree

M: male; F: female.

**Table 2:** RDC/TMD diagnosis at baseline and follow-ups (1 and 6 months).

Patient	Research diagnostic criteria					
	Axis I					
	Group I		Group II		Group III	
			Right	Left	Right	Left
1	Baseline	MPWLO	ADDR	ADDR	—	—
	Follow-up (1 month)	—	ADDR	ADDR	—	—
	Follow-up (6 months)	—	ADDR	ADDR	—	—

2	Baseline	—	ADDR	ADDR	—	—
	Follow-up (1 month)	—	ADDR	ADDR	—	—
	Follow-up (6 months)	—	ADDR	ADDR	—	—

3	Baseline	—	ADDR	ADDR	—	—
	Follow-up (1 month)	—	—	—	—	—
	Follow-up (6 months)	—	—	—	—	—

4	Baseline	—	ADDR	ADDR	—	—
	Follow-up (1 month)	—	ADDR	ADDR	—	—
	Follow-up (6 months)	—	ADDR	ADDR	—	—

5	Baseline	MP	ADDR	ADDR	Arthralgia	Arthralgia
	Follow-up (1 month)	—	ADDR	ADDR	—	—
	Follow-up (6 months)	—	ADDR	ADDR	—	—

6	Baseline	MP	ADDR	ADDR	Osteoarthritis	Osteoarthritis
	Follow-up (1 month)	—	ADDR	ADDR	Osteoarthritis	Osteoarthrosis
	Follow-up (6 months)	—	ADDR	ADDR	Osteoarthritis	Osteoarthrosis

7	Baseline	MP	ADDR	ADDR	—	—
	Follow-up (1 month)	—	ADDR	ADDR	—	—
	Follow-up (6 months)	—	ADDR	ADDR	—	—

8	Baseline	—	ADDR	ADDR	—	—
	Follow-up (1 month)	—	ADDR	ADDR	—	—
	Follow-up (6 months)	—	ADDR	ADDR	—	—

9	Baseline	—	—	—	Osteoarthrosis	Osteoarthritis
	Follow-up (1 month)	—	—	—	Osteoarthrosis	Osteoarthritis
	Follow-up (6 months)	^*∗*^	^*∗*^	^*∗*^	^*∗*^	^*∗*^

10	Baseline	MPWLO	ADDR	ADDR	—	—
	Follow-up (1 month)	—	ADDR	ADDR	—	—
	Follow-up (6 months)	^*∗*^	^*∗*^	^*∗*^	^*∗*^	^*∗*^

RDC/TMD Axis I Group I (muscle disorders): MP = myofascial pain, MPWLO = myofascial pain with limited opening; Group II (disc displacement): ADDR = disc displacement with reduction; Group III (other joint conditions). ^*∗*^Patient did not attend final follow-up.

**Table 3:** CBCT evaluation of osteoarthritis changes at baseline and at final (6 months) follow-up.

Patient		Osteoarthritis changes of TMJ (mm)								
		Sclerosis		Erosion		Osteophyte			Flattening	
		Right joint	Left joint	Right joint	Left joint	Right joint	Left joint	Right joint		Left joint
2	Baseline	2.370	1.270	1.410	0.420	1.580	0.000	4.510		0.000
	Final	1.020	1.220	0.410	0.290	1.040	0.000	3.130		0.000

3	Baseline	1.210	1.630	0.000	0.000	0.590	0.000	4.950		4.620
	Final	1.060	0.870	0.000	0.000	0.510	0.000	2.220		2.160

4	Baseline	1.800	1.400	0.000	0.000	0.000	0.000	4.070		2.910
	Final	1.280	1.100	0.000	0.000	0.000	0.000	2.000		2.470

5	Baseline	2.470	1.960	1.080	0.730	1.870	1.190	5.570		4.560
	Final	1.550	1.950	0.850	0.350	1.300	0.850	2.520		2.220

6	Baseline	1.610	1.520	0.730	0.000	2.230	0.000	6.380		3.480
	Final	1.560	1.030	0.420	0.000	1.110	0.000	2.410		3.190

7	Baseline	1.020	1.090	0.000	0.550	1.240	1.220	3.250		3.620
	Final	0.920	0.770	0.000	0.190	1.030	0.770	1.650		3.300

8	Baseline	0.880	0.680	0.000	0.000	0.430	0.430	0.460		4.140
	Final	0.690	1.630	0.000	0.000	0.000	0.410	2.220		4.110

Baseline	Mean or median	1.460		0.340		0.510			4.105	
	SD	—		0.470		—			—	
	25%	1.120		—		0.000			3.300	
	75%	1.750		—		1.230			4.600	

Final	Mean or median	1.140		0.170		0.460			2.440	
	SD	—		0.250		—			—	
	25%	0.940		—		0.000			2.220	
	75%	1.550		—		0.980			3.170	

*P* value	Paired *t*-test			0.022^*∗*^						
	Wilcoxon test	0.041^*∗*^				0.00^*∗*^			0.027^*∗*^	

Media and standard deviation (SD) are shown for erosion (parametric data). Median, 25th percentile (25%), and 75th percentile (75%) are shown for other parameteres (nonparametric data); *p* values of 0.05 or less were considered significant.

**Table 4:** MRI evaluation of TMJ disc position and adhesion at baseline and at final (6 months) follow-up.

Patient		TMJ soft tissues evaluation							
		Right joint				Left joint			
		Sagittal plane^*∗*^	Coronal plane^*∗*^	Adhesion	Reduction	Sagittal plane^*∗*^	Coronal plane^*∗*^	Adhesion	Reduction
2	Baseline	AI	S	No	Yes	A	Lateral	No	Yes
	Final	AI	S	No	Yes	A	S	No	Yes

3	Baseline	S	Medial	Yes	Yes	S	S	No	Yes
	Final	A	S	No	Yes	A	S	No	Yes

4	Baseline	A	S	No	Yes	AI	S	No	No
	Final	A	S	No	Yes	S	S	No	Yes

5	Baseline	A	S	No	No	AI	Lateral	No	No
	Final	A	S	No	Yes	A	S	No	Yes

6	Baseline	A	Lateral	No	Yes	A	S	No	Yes
	Final	A	S	No	Yes	AI	S	No	Yes

7	Baseline	A	S	No	Yes	S	S	No	Yes
	Final	S	S	No	Yes	S	Lateral	No	Yes

8	Baseline	A	Lateral	No	Yes	A	S	No	Yes
	Final	A	S	No	No	A	S	No	Yes

^*∗*^Position of disc posterior band to functional surface of the mandibular head in sagittal and coronal planes: S: superior; A: anterior; AI: anteroinferior. Two patients did not attend final follow-up and 1 could not be submitted to CBCT or MRI because of pregnancy.

## Data Availability

The data used to support the findings of this study are included within the article.
